# Responses of Nitrogen Metabolism, Uptake and Translocation of Maize to Waterlogging at Different Growth Stages

**DOI:** 10.3389/fpls.2017.01216

**Published:** 2017-07-11

**Authors:** Baizhao Ren, Shuting Dong, Bin Zhao, Peng Liu, Jiwang Zhang

**Affiliations:** State Key Laboratory of Crop Biology and College of Agronomy, Shandong Agricultural University Taian, China

**Keywords:** maize (*Zea mays* L.), waterlogging in the field, N metabolism, N uptake and translocation, grain yield

## Abstract

We performed a field experiment using the maize hybrids DengHai605 (DH605) and ZhengDan958 (ZD958) to study nitrogen uptake and translocation, key enzyme activities of nitrogen metabolism in response to waterlogging at the third leaf stage (V3), the sixth leaf stage (V6), and the 10th day after the tasseling stage (10VT). Results showed that N accumulation amount was significantly reduced after waterlogging, most greatly in the V3 waterlogging treatment (V3-W), with decreases of 41 and 37% in DH605 and ZD958, respectively. N accumulation in each organ and N allocation proportions in grains decreased significantly after waterlogging, whereas N allocation proportions increased in stem and leaf. The reduction in stem and leaf N accumulation after waterlogging was mainly caused by a decrease in dry matter accumulation, and a reduction in N translocation from stems and leaves to grains after waterlogging. Additionally, waterlogging decreased the activity of key N metabolism enzymes (nitrate reductase, glutamine, glutamate synthase, and glutamate dehydrogenase), and the most significant reduction in V3-W with a decrease of 59, 46, 35, and 26% for DH605, and 60, 53, 31, and 25 for ZD958, respectively. Waterlogging disrupted N metabolism, hindered N absorption and transportation, and decreased maize yield.

## Introduction

Waterlogging is one of major abiotic stresses in crop production. Globally, it is estimated that 12% of cropping areas are affected by waterlogging (Sergey, 2011). By influencing crop growth and development (Kozlowski, 1997; Jackson and Campbell, 2006), waterlogging significantly reduces grain yield. In the Huanghuaihai Plain, most rainfall takes place during maize growth periods; thus, waterlogging is a frequent natural disturbance to maize production (Chi and Zhou, 2006) which inhibits its growth and development (Ren et al., 2014a). In waterlogged soil, anaerobic respiration is enhanced, a large number of harmful substances, such as hydrogen sulfide (H_2_S) and ferrous sulfide (FeS) accumulate, and the rhizosphere environment deteriorates leading to inhibited absorption of mineral ions and beneficial trace elements, and eventually resulting in disruption of root growth and development (Ashraf and Rehman, 1999; Przywara and Stcdaniewski, 1999). The restriction of root growth, induced by waterlogging, limits the absorption of nitrogen (N) fertilizer absorption, disrupting N uptake, transportation, and distribution in each organ, eventually leading to a reduction in N use efficiency (Ren et al., 2016).

The inhibition of crop growth after waterlogging may be caused by soil oxygen deficit, which inhibits the main metabolism of crops and changes the effectiveness of crop nutrition and soil nutrients (Lawlor, 2002; Milroy et al., 2009; Ren et al., 2016). N metabolism is a basic physiological process in which related enzymes play an important role in plant resistance to adverse environmental conditions (Ramanjulu and Sudhakar, 1997). N metabolism has been shown to contribute cellular acclimation to low oxygen stress in plants (Bailey-Serres et al., 2012). N deficiency may be induced by the low redox potential in waterlogged soils that promotes denitrification of nitrate (NO_3_^-^) (Limami et al., 2014). Nitrate reductase (NR), glutamine (GS), glutamate synthase (GOGAT), and glutamate dehydrogenase (GDH) are key enzymes in N metabolism, whose activities have been used as representative biochemical markers to evaluate N status. NR is a key enzyme which adjusts the N assimilation and metabolism process, and is sensitive to changes in environmental conditions (Kaiser and Huber, 1994). The reduction of NR activity in leaves of waterlogged plants is due to a rapid depletion of NO_3_^-^ and oxygen under anaerobic conditions (Sung and Sun, 1990; Hoff et al., 1992). As a result, volatilization and loss of NO_3_^-^are promoted through denitrification. Under water stress, NR activity can be suppressed, limiting the reduction of NO_3_^-^ and the assimilation of NH_4_^+^, leading to the generation of NH_4_^+^ and a short supply of alpha ketone glutaric acid; thus activities of GS and GOGAT are reduced, resulting in the accumulation of NH_4_^+^In recent years, much research has been conducted on the responses of NR, GS, GOGAT, and GDH activities in crops to various environmental stresses (Eva et al., 2011; Robredo et al., 2011) and cultivation methods (Debouba et al., 2006; Monreal et al., 2007). However, very little attention has been given to the effects of waterlogging on N metabolism, N uptake, and translocation in maize. To examine the responses of N metabolism, uptake and translocation of maize to waterlogging at different stages, we performed a field experiment in which we determined the effects of waterlogging at the third leaf stage, the sixth leaf stage, and the 10th day after the tasseling stage on N accumulation and translation, the activities of N metabolism enzymes, and yield.

## Materials and Methods

### Experimental Location

A field experiment was conducted at the State Key Laboratory of Crop Biology and the experimental farm of Shandong Agricultural University, China (36°10′N, 117°04′E, 151 m altitude) in 2012 and 2013. The region has a temperate continental monsoon climate. The effective accumulated temperatures of maize growth periods in 2012 and 2013 were 1710.9°C d and 1740.5°C d, respectively. The mean total precipitation values during maize growth periods in 2012 and 2013 were 350.0 mm and 348.5 mm, respectively. The soil type was sandy loam, and soil pH was 8.25 (Cambisols; FAO/EC/ISRIC 2003). The plowed soil (0–20 cm) before the experiment contained 10.2 g kg^-1^ of organic matter, with total mounts of N: 0.9 g kg^-1^, rapidly available phosphorus (P): 50.3 mg kg^-1^, and rapidly available potassium (K): 85.4 mg kg^-1^.

### Experimental Design

Each plot was 4 m × 4 m and separated by 4 m × 2.3 m polyvinyl chloride (PVC) boards as water barriers. Every PVC board was buried 2.0 m below the surface, with remaining 0.3 m aboveground. Experimental treatments matched different waterlogging stages: the third leaf stage (V3; V3-W), the sixth leaf stage (V6; V6-W), and the 10th day after the tasseling stage (10VT; 10VT-W), and no waterlogging (CK). In the CK, soil moisture was kept optimum during the whole growth period. We selected the maize hybrids DengHai605 (DH605) and ZhengDan958 (ZD958) as experimental materials, because they are the most commonly planted varieties in China. Maize was sown on June 16 in both years, at a plant density of 67,500 plants ha^-1^. The water in waterlogged pools was maintained at 2–3 cm above the soil surface for 6 d. Each treatment was replicated three times, in a completely randomized block design. 300 kg ha^-1^ urea (N 46%), 857 kg ha^-1^ calcium superphosphate (P_2_O_5_ 17%), and 400 kg ha^-1^ muriate of potash (K_2_O 60%) were applied. Prior to seeding, P, K compound fertilizer was applied one-off to prepare the soil for sowing, 40% N compound fertilizer was applied at V6, and 60% N compound fertilizer was applied at V12. Disease, weeds, and pests were well controlled in each treatment.

### Dry Matter and N Amount

Five representative plant samples were obtained from each plot at V6, the twelfth leaf stage (V12), the tasseling stage (VT), the milk stage (R3), and the physiological maturity stage (R6), according to Ritchie and Hanway (1982). Samples were preserved after being separated into stem and leaf at V6, V12, and VT, and separated into stem, leaf, and ear at R3 and R6. Samples were dried at 80°C in a force-draft oven (DHG-9420A, Bilon Instruments Co., Ltd, Shanghai, China) to a constant weight and weighed separately. Total N was measured using the Kjeldahl method (Hibbard, 2002):

$$\begin{array}{c} \mathrm{Nitrogen}\mathrm{harvest}\mathrm{index}(NHI,\%) =\mathrm{grain}Namount/totalN\mathrm{amount}\mathrm{of}\mathrm{plant}\\ \;\mathrm{Harvest}\mathrm{index}(HI,\%) =\mathrm{grain}\mathrm{dry}weight/total\mathrm{dry}\mathrm{weight}\mathrm{of}\mathrm{plant} \end{array}$$

### NR Activity

The functional leaves from five plant samples were obtained from the center of each plot at the next day after the end of waterlogging treatments. NR activity was estimated using the method of Aslam et al. (2001). The samples (0.5 g) were placed in 10 mL of incubation medium, which was 0.1 M potassium phosphate buffer (pH 7.5) containing 0.1 M KNO_3_ with 1% (v/v) propanol. Prior to the assay, the buffer solution was purged with N_2_ gas for 30 min to remove dissolved oxygen, and samples then were vacuum-infiltrated (two times), and incubated in a water bath at 30°C for 30 min in the dark. 1 mL sample was withdrawn for the color reaction, and initiated by adding 2 mL aminobenzenesulfonic acid and 2 mL a-naphthylamine. After 20 min of incubation, the amount of nitrite (NO_2_^-^) was determined by absorbance at 520 nm using a standard curve.

### GS, GOGAT, and GDH Activities

The functional leaves from five plant samples were obtained from the center of each plot at the next day after the end of waterlogging treatments. To extract enzymes, 0.5 g of leaf tissue was homogenized with 10 mM Tris-HCl buffer (pH 7.6, containing 1mM MgCl_2_, 1 mM EDTA, and 1mM 2-mercaptoethanol) in a chilled pestle and mortar. The homogenate was centrifuged at 15000 × *g* for 30 min at 4°C. The supernatant was used to determine enzyme activities.

Glutamine was assayed according to Mohanty and Fletcher (1980). The reaction mixture contained in a final volume of 1 mL, 80 μmol Tris-HCl buffer, 40 μmol L-glutamic acid, 8 μmol ATP, 24 μmol MgSO_4_, and 16 μmol NH_2_OH. The final pH was 8.0. The reaction was initiated by the addition of the enzyme extract. After incubation for 30 min at 30°C, the reaction was stopped by adding 2 mL 2.5% (w/v) FeCl_3_ and 5% (w/v) trichloroacetic acid in 1.5 M HCl. After centrifugation at 3000 × *g* for 10 min, the absorbance of the supernatant was read at 540 nm. GS activity was expressed as 1 μmol L-glutamate γ-monohydroxamate (GHA) formed g^-1^ FW h^-1^, with μmol GHA g^-1^ FM h^-1^ said.

Glutamate synthase activity was measured based on the method described by Singh and Srivastava (1986), in units of μmol NADH g^-1^ FM min^-1^. The assay mixture contained 0.4 mL 20 mM L-glutamine, 0.05 mL 0.1 M 2-oxoglutarate, 0.1 mL 10 mM KCI, 0.2 mL 3 mM NADH, and 0.5 mL of the enzyme extract in a final volume of 3 mL, prepared with 25 mM Tris-HCl buffer (pH 7.6). The reaction was initiated by adding L-glutamine immediately following the enzyme preparation. The decrease in absorbance was recorded for 3 min at 340 nm.

Glutamate dehydrogenase activity was estimated by using the method of Lin and Kao (1996), in unites of μmol NADH g^-1^ FM min^-1^. The assay mixture contained 0.3 mL 0.1 M 2-oxoglutarate, 0.3 mL 1 M NH_4_Cl, 0.2 mL 3 mM NADH and 1 mL of the enzyme extract in a final volume of 3 mL, prepared with 0.2 M Tris-HCl buffer (pH 8.0). The reaction was initiated by adding the enzyme extract. The decrease in absorbance was recorded for 3 min at 340 nm.

### Yield

At R6, 30 ears harvested from three rows at the center of each plot were used to determine yield and ear traits including length, width, weight, row number, kernels per row, bald tip length, cob weight, and cob width. All kernels were air-dried to determine yield, and grain yield was expressed at 14% moisture content, according to the standard moisture content of maize for storage or sale is 14% in China (GB/T 29890-2013).

$$\begin{array}{c} Grain yield(kg{\mathrm{ha}}^{-}{}^{1}) = Harvest ear(ears{\mathrm{ha}}^{-}{}^{1}) \times kernel number per ear \times 1000-grain weight(g{1000grains}^{-}{}^{1}){/10}^{6}{}^{\;}\times (1 - moisture content\%)/(1 - 14\%) \end{array}$$

### Statistical Analysis

The data were subjected to three-way analysis of variance (ANOVA). Growing season, blocks, and block interactions were included as random effects. Waterlogging treatment and hybrids were included as fixed effects. In case of significant treatment effects, comparison of means was performed by means of LSD at a significance level of 0.05. LSD was used to compare adjacent means arranged in order of magnitude. ANOVA and the LSD test were conducted using the SPSS17.0 software program (Ver. 17.0, SPSS, Chicago, IL, United States). Figures were prepared using a SigmaPlot 10.0 program.

## Results

### N Uptake and Translocation

**FIGURE 1:**
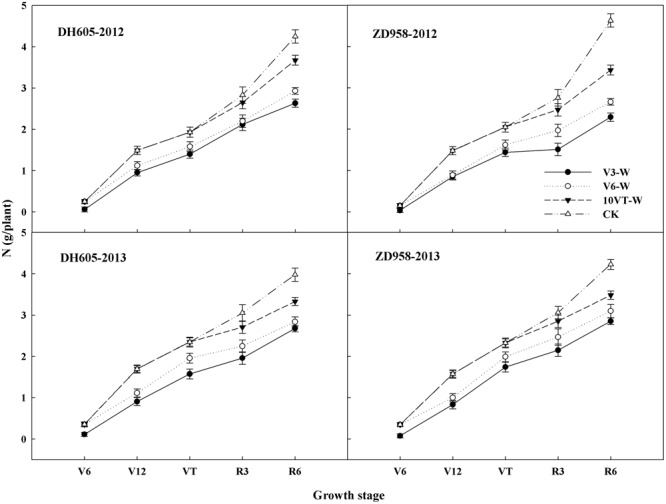
Effects of waterlogging on the N accumulation of maize. V3-W: waterlogging at the third leaf stage; V6-W: waterlogging at the sixth leaf stage; 10VT-W: waterlogging at 10th day after the tasseling stage. Means and standard errors based on three replicates are shown.

Waterlogging significantly affected N uptake and translocation in maize. The V3 stage was most susceptible to waterlogging, followed by V6 and 10VT stages. Waterlogging significantly decreased total N accumulation in maize, compared to CK. The total N accumulation levels of DH605 in treatments V3-W, V6-W, and 10VT-W were 39, 36, and 19% lower than that of CK across hybrids and years (**Figure 1**). There were no significant year × hybrid × waterlogging treatment interaction effects on N accumulation and distribution at the R6 stage (**Table 1**). The greatest reduction sin stem, leaf, cob, and grain N accumulation were found in the V3-W treatment at 25, 18, 34, and 46% across hybrids and years, compared to those in CK. Additionally, waterlogging increased N distribution rate in leaf, stem, and cob, whereas grain N distribution rate decreased significantly after waterlogging, compared to CK. Waterlogging significantly decreased NHI of maize, with the most significant reduction (approximately 10%) in the V3-W treatment across hybrids and years, compared to that of CK (**Table 1**).

**Table 1:** Effect of waterlogging on N accumulation (g plant^-1^) and distribution (%) of maize at maturity stage.

Year (Y)	Hybrid (H)	Treatment (T)	Total accumulation	Stem		Leaf		Cob		Grain		NHI
			(g plant^-1^)	g plant^-1^	%	g plant^-1^	%	g plant^-1^	%	g plant^-1^	%	
2012	DH605	V3-W	2.02c	0.26c	12.8	0.34b	16.7	0.06b	3.0	1.36c	67.6	0.68c
		V6-W	2.16c	0.27bc	12.5	0.35b	16.3	0.06b	2.9	1.48c	68.2	0.68c
		10VT-W	2.93b	0.30ab	10.2	0.40a	13.5	0.09ab	3.2	2.14b	73.2	0.73b
		CK	4.08a	0.32a	7.8	0.42a	10.4	0.13a	3.1	3.22a	78.7	0.79a
	ZD958	V3-W	2.22c	0.20c	8.9	0.43c	19.4	0.13b	5.7	1.46c	66	0.66c
		V6-W	2.34c	0.26b	11.1	0.45bc	19.5	0.14b	5.9	1.48c	63.6	0.64c
		10VT-W	3.24b	0.27ab	8.3	0.47b	14.5	0.19a	5.7	2.32b	71.5	0.72b
		CK	3.88a	0.29a	7.4	0.53a	13.7	0.21a	5.4	2.85a	73.5	0.74a
2013	DH605	V3-W	2.85c	0.30c	10.5	0.43b	15.1	0.12b	4.2	2.00d	70.2	0.70b
		V6-W	3.09bc	0.32bc	10.4	0.46b	15	0.12b	4.0	2.19c	70.9	0.71b
		10VT-W	3.48b	0.33b	9.6	0.55a	15.7	0.13ab	3.8	2.47b	71.0	0.71b
		CK	4.22a	0.40a	9.4	0.58a	13.9	0.14a	3.4	3.10a	73.4	0.73a
	ZD958	V3-W	2.67c	0.27b	10.2	0.48b	18.0	0.15b	6.4	1.78c	66.7	0.67b
		V6-W	2.83bc	0.30b	10.5	0.49ab	17.2	0.15b	5.3	1.89c	66.8	0.67b
		10VT-W	3.32ab	0.30b	9.1	0.50ab	15.1	0.17b	5.1	2.24b	67.5	0.67b
		CK	3.97a	0.35a	8.8	0.51a	12.9	0.20a	5.1	2.90a	73.1	0.73a
	ANOVA											
	Y		^∗∗^	^∗∗^		^∗^		^∗^		^∗^		NS
	H		NS	^∗∗^		NS		^∗∗^		NS		^∗^
	T		^∗∗^	^∗∗^		NS		^∗∗^		^∗∗^		^∗^
	Y × H		^∗^	NS		^∗^		^∗^		NS		NS
	Y × T		NS	NS		NS		NS		NS		NS
	H × T		NS	NS		NS		NS		NS		NS
	Y × H × T		NS	NS		NS		NS		NS		NS

*V3-W, waterlogging at the third leaf stage; V6-W, waterlogging at the sixth leaf stage; 10VT-W, waterlogging at 10th day after the tasseling stage; NHI, N harvest index. Values fallowed by a different small letter within a column are significantly different at 5% probability level. Differences between treatments were calculated within the hybrids for each particular year. NS, Not significant. ^∗^Significant at the 0.05 probability level. ^∗∗^Significant at the 0.01 probability level.*

### N Metabolism Enzyme Activity

Waterlogging significantly decreased the activities of key N metabolism enzymes, compared to CK, with no significant hybrid × waterlogging treatment interaction effects (**Table 2**). The greatest reduction in the activities of NR (approximately 60%), GS (approximately 50%), GDH (approximately 33%), and GOGAT (approximately 26%) took place when waterlogging occurred at V3, whereas waterlogging at V6 produced activity reductions of 37, 47, 25, and 20%, and waterlogging at 10VT produced activity reductions of 11, 29, 17, and 16% for these key N metabolism enzymes across hybrids and years.

**Table 2:** Effect of waterlogging on NR (μg g^-1^ FM h^-1^), GS (μmol GHA g^-1^ FM h^-1^), GDH (μmol NADH g^-1^ FM min^-1^), GOGAT (μmol NADH g^-1^ FM min^-1^) activities of maize (2013).

Waterlogging periods	Hybrid	Treatment	NR	GS	GDH	GOGAT
V3	DH605	T	6.8b	3.3b	70.3b	65.6b
		CK	16.5a	6.1a	107.7a	89.0a
		±CK%	–58.8	–45.6	–34.7	–26.2
	ZD958	T	7.6b	3.9b	74.1b	74.4b
		CK	18.9a	8.3a	107.2a	98.7a
		±CK%	–59.8	–53.3	–30.9	–24.6
V6	DH605	T	28.9b	8.3b	123.8b	113.1b
		CK	46.6a	14.8a	174.4a	140.2a
		±CK%	–38.0	–43.7	–29.0	–19.3
	ZD958	T	38.6b	3.9b	146.6b	108.0b
		CK	58.9a	7.6a	186.2a	138.0a
		±CK%	–34.5	–48.8	–21.3	–21.7
10VT	DH605	T	28.9b	7.5b	97.0b	87.3b
		CK	32.5a	10.5a	113.6a	105.1a
		±CK%	–11.1	–28.7	–14.6	–16.9
	ZD958	T	27.5b	7.5b	88.9b	85.5b
		CK	30.9a	10.4a	109.3a	100.9a
		± CK%	–11.0	–28.2	–18.7	–15.3
	ANOVA					
	Hybrid (H)		NS	NS	ˆ*	NS
	Treatment (T)		ˆ**	ˆ*	ˆ*	ˆ*
	H × T		NS	NS	ˆ*	NS

*V3-W, waterlogging at the third leaf stage; V6-W, waterlogging at the sixth leaf stage; 10VT-W, waterlogging at 10th day after the tasseling stage; NR, nitrate reductase; GS, glutamine; GOGAT, glutamate synthase; GDH, glutamate dehydrogenase. Values fallowed by a different small letter within a column are significantly different at 5%probability level. Differences between treatments were calculated within the hybrids. NS, Not significant. ^∗^Significant at the 0.05 probability level. ^∗∗^Significant at the 0.01 probability level.*

### Dry Matter Accumulation and Distribution

**FIGURE 2:**
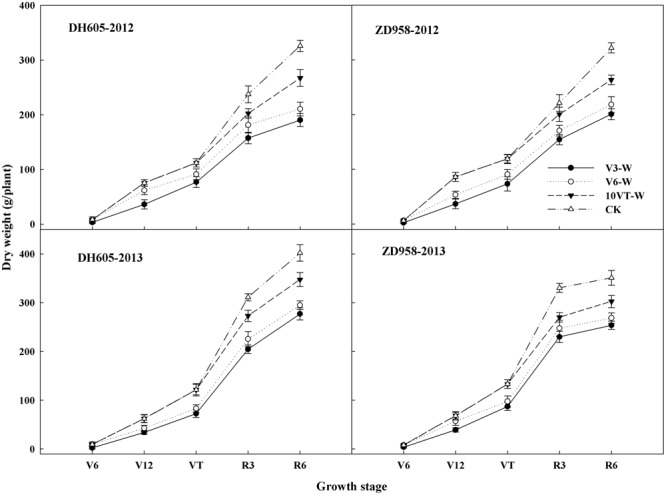
Effects of waterlogging on dry matter accumulation of maize. V3-W: waterlogging at the third leaf stage; V6-W: waterlogging at the sixth leaf stage; 10VT-W: waterlogging at 10th day after the tasseling stage. Means and standard errors based on three replicates are shown.

Effects of waterlogging on dry matter accumulation were similar between the two hybrids (**Figure 2**). Total dry matter accumulation was significantly reduced by waterlogging at different stages, with the most significant reduction (approximately 34%) in the V3-W treatment across hybrids and years. There were no significant year × hybrid × waterlogging treatment interaction effects on dry matter accumulation or distribution at the R6 stage (**Table 3**). The greatest reduction in dry matter weight in stem (approximately 22%), leaf (approximately 27%), cob (approximately 21%), and grain (approximately 42%) occurred in the V3-W treatment. Waterlogging increased dry matter distribution rate in stem, leaf, and cob, whereas that in grain was significantly reduced after waterlogging. Waterlogging also significantly decreased the HI, with the most significant reduction (approximately 11%) in V3-W across hybrids and years, compared to that of CK (**Table 3**).

**Table 3:** Effect of waterlogging on dry matter accumulation (g plant^-1^) and distribution (%) of maize at maturity stage.

Year (Y)	Hybrid (H)	Treatment (T)	Total dry matter (g plant^-1^)	Stem		Leaf		Cob		Grain		HI
				g plant^-1^	%	g plant^-1^	%	g plant^-1^	%	g plant^-1^	%	
2012	DH605	V3-W	203c	46b	22.5	25b	12.6	19b	9.4	112d	55.5	0.56c
		V6-W	219c	49b	22.3	27b	12.3	19b	8.8	124c	56.5	0.57bc
		10VT-W	264b	57a	21.7	32a	12.0	22ab	8.4	153b	58.0	0.58b
		CK	321a	62a	19.1	35a	10.9	26a	8.2	199a	61.8	0.62a
	ZD958	V3-W	192c	46c	24.0	25b	12.9	23c	11.9	98c	51.3	0.51c
		V6-W	211c	50c	23.9	27b	12.8	24bc	11.3	110c	52.1	0.52bc
		10VT-W	267b	59b	22.2	34a	12.7	27ab	10.1	147b	55.0	0.55b
		CK	326a	64a	19.5	36a	11.1	30a	9.2	196a	60.2	0.60a
2013	DH605	V3-W	277c	78c	28.1	32c	11.4	26b	9.5	142c	51.1	0.51b
		V6-W	285c	80c	28.2	29bc	10.3	27b	9.3	147c	51.5	0.52b
		10VT-W	347b	88b	25.4	42ab	12.0	27b	7.8	190b	54.8	0.55a
		CK	402a	96a	24.0	43a	10.8	32a	8.0	230a	57.3	0.57a
	ZD958	V3-W	254c	80s	31.7	30c	12.0	22b	8.7	122d	48.1	0.48c
		V6-W	268c	85c	31.5	31c	11.5	22b	8.2	131c	48.7	0.49b
		10VT-W	303b	90b	29.8	34b	11.1	24ab	8.1	154b	51.0	0.51ab
		CK	351a	97a	27.6	38a	10.8	26a	7.5	189a	53.9	0.53a
	ANOVA											
	Y		^∗∗^	^∗∗^		^∗^		^∗∗^		^∗^		^∗∗^
	H		NS	^∗^		NS		NS		^∗^		^∗∗^
	T		^∗∗^	^∗∗^		^∗^		^∗∗^		^∗∗^		^∗∗^
	Y × H		NS	NS		NS		^∗∗^		NS		NS
	Y × T		NS	NS		NS		NS		NS		NS
	H × T		NS	NS		NS		NS		NS		NS
	Y × H × T		NS	NS		NS		NS		NS		NS

*V3-W, waterlogging at the third leaf stage; V6-W, waterlogging at the sixth leaf stage; 10VT-W, waterlogging at 10th day after the tasseling stage; HI, harvest index. Values fallowed by a different small letter within a column are significantly different at 5% probability level. Differences between treatments were calculated within the hybrids for each particular year. NS, Not significant. ^∗^Significant at the 0.05 probability level. ^∗∗^Significant at the 0.01 probability level.*

### Grain Yield

Waterlogging resulted in a significant yield reduction in maize (**Table 4**). The greatest yield reduction (approximately 38%) occurred in the V3, whereas waterlogging at V6 and 10VT produced yield reductions of 30 and 15% across hybrids and years, respectively. Waterlogging also decreased grain number per ear and 1000-grain weight, with the most significant reduction in the V3-W treatment with decreases of 22 and 16% across hybrids and years.

**Table 4:** Effects of waterlogging on grain yield and yield components of maize.

Year	Hybrid	Treatment	Ear number	Grain number per grain	1000-grain weight (g)	Grain yield (kg ha^-1^)
2012	DH605	V3-W	61,714	438c	354d	9512d
		V6-W	61,822	459d	366c	10591c
		10VT-W	63,851	480b	373b	11437b
		CK	65,642	530a	403a	14018a
	ZD958	V3-W	61,192	453d	322c	9012d
		V6-W	62,100	481c	317c	9292d
		10VT-W	63,367	543b	349b	12173b
		CK	66,250	573a	366a	13910a
2013	DH605	V3-W	65,421	395d	321d	8310d
		V6-W	65,607	453c	351c	10431c
		10VT-W	65,590	495b	371b	12043b
		CK	65,607	556a	389a	14207a
	ZD958	V3-W	63,679	421d	273d	7314d
		V6-W	63,355	446c	294c	8312c
		10VT-W	65,625	521b	321b	10999b
		CK	66,875	537a	345a	12388a
	ANOVA					
	Year (Y)		NS	NS	^∗∗^	^∗^
	Hybrid (H)		NS	NS	^∗∗^	^∗^
	Treatment (T)		^∗^	^∗^	^∗∗^	^∗∗^
	Y × H		NS	NS	^∗^	^∗^
	Y × T		NS	NS	NS	NS
	H × T		NS	NS	NS	NS
	Y × H × T		NS	NS	NS	NS

*V3-W, waterlogging at the third leaf stage; V6-W, waterlogging at the sixth leaf stage; 10VT-W, waterlogging at 10th day after the tasseling stage. Values fallowed by a different small letter within a column are significantly different at 5%probability level. Differences between treatments were calculated within the hybrids for each particular year. NS, Not significant. ^∗^Significant at the 0.05 probability level. ^∗∗^Significant at the 0.01 probability level.*

## Discussion

N is a key plant nutrient and signal molecule which controls many aspects of plant metabolism and development (Stitt et al., 2002; Krouk et al., 2010). Previous studies showed that waterlogging significantly affects plant nutrient accumulation and distribution (Iqra and Naveela, 2013). Our study also showed that waterlogging at different stages significantly decreased N accumulation of each organ. However, waterlogging increased N distribution rate in stem and leaf, whereas grain N distribution rate decreased significantly after waterlogging. These results were in agreement with previous studies (Ren et al., 2014b; Phukan et al., 2016). Photosynthetic capacity of plant is closely associated with leaf N (Jiang et al., 2004). However, our results showed that waterlogging inhibited the accumulation of leaf N in maize, which would limit photosynthetic capacity, and thus decrease plant photosynthesis and dry-matter accumulation (Mu et al., 2016), ultimately resulting in the disruption of dry matter accumulation and translation in maize (**Figure 2** and **Table 3**). The reduction in leaf nutrients induced by waterlogging lead to a reduction in “sink” characteristics and affected the normal “source” characteristics of photosynthetic and grain filling, resulting in a significant reduction in grain weight and yield (Ren et al., 2014a). Additionally, nitrogen harvest index (NHI) reflects N distribution in grain and vegetative organs at R6 stage (Jin et al., 2012). However, waterlogging decreases significantly grain N accumulation, resulting in the reduction of the NHI (Limami et al., 2014). Our study also showed waterlogging significantly decreased the NHI in maize, indicating that waterlogging significantly decreased grain N accumulation, and affected N use efficiency in maize. The most significant inhibition of NHI induced by waterlogging was observed at V3, followed by V6 and 10VT.

Waterlogging decreased leaf N accumulation, indicating that waterlogging inhibited N metabolism and assimilation, and disrupted crop physiological function. NR is one of key N metabolism enzymes, which controls the first step of N uptake and utilization, catalyzing the conversion of NO_3_^-^ into NO_2_^-^, and is significantly positively related to corn production (Singletary et al., 1990). NR is also a photoinduced enzyme whose activity is easily affected by environmental factors such as light, temperature, and moisture (Tischner, 2000). Previous study (Phukan et al., 2016) has shown that waterlogging does lead to decrease of NR activity in plant leaves, resulting in a significant reduction of N use efficiency. In our study, NR activity in maize was significantly decreased by waterlogging, indicating that waterlogging inhibited N metabolism, and disrupted N uptake and translation. The coupled GS-GOGAT reaction cycle is the main channel of N metabolism (Miflin and Habash, 2002). GS activity is significantly positively related to protein hydrolysis and the ability to adapt to abiotic stress (Dwivedi et al., 2012). Our study showed that GS and GOGAT activities declined significantly after waterlogging, leading to a significant drop in the activities of N metabolism enzymes and sugar metabolism enzymes in maize leaves, affecting the synthesis and transformation of amino acids (Limami et al., 2014), and ultimately inhibiting N metabolism, and disrupting N absorption and translation; thus, physiological processes associated with N became limited, resulting in a significant yield reduction of maize. GDH plays an important role in protein synthesis in the late grain-filling stage, and participated in the resynthesis of NH_4_^+^ under environmental stresses. The importance of GDH in the control of N assimilation and recycling of rice (Yamaya et al., 2002) and maize (Gallais and Hirel, 2004) has been established using physiological and quantitative genetic approaches; it is important in putative key reactions influencing grain yield and its components (Hirel et al., 2001; Dubois et al., 2003). Our study showed that waterlogging decreased GDH activity. This result indicated that waterlogging inhibited N assimilation and recycling, resulting in reduced grain yield. The NR, GS, GDH, and GOGAT activities of maize were significantly decreased after waterlogging, indicating that waterlogging inhibited leaf normal N metabolism, resulting in reduced N use efficiency, thus limiting normal physiological function associated with N. N metabolism was most susceptible to damage when waterlogging occurred at the V3 stage, followed by V6 and 10VT stages.

## Conclusion

Waterlogging decreased the activity of key N metabolism enzymes (nitrate reductase, glutamine, GOGAT, and GDH), resulting in the inhibition of leaf normal N metabolism. Waterlogging also hindered N accumulation and translation, and reduced maize yield. The V3 stage was most susceptible to waterlogging, followed by V6 and 10VT stages.

## Author Contributions

JZ and BR initiated and designed the research. BR performed the experiments, analyzed the data, and wrote the manuscript. JZ, SD, PL, and BZ revised and edited the manuscript and also provided advice on the experiments.

## Conflict of Interest Statement

The authors declare that the research was conducted in the absence of any commercial or financial relationships that could be construed as a potential conflict of interest.
